# Modeling Vehicle Dust Extraction Impeller Degradation Using TOPSIS-Selected Optimal Degradation Trajectory

**DOI:** 10.3390/ma19132910

**Published:** 2026-07-07

**Authors:** Feng Zhang, Xunhao Zhang, Jinze Liu, Xue Li, Ruiyang Zhang, Yuxiang Tian

**Affiliations:** School of Mechanics and Transportation Engineering, Northwestern Polytechnical University, Xi’an 710129, Chinazhry@mail.nwpu.edu.cn (R.Z.);

**Keywords:** dust extraction impeller, erosion wear, TOPSIS, accelerated degradation test, degradation modeling

## Abstract

The dust extraction impeller is a core component of the vehicle engine auxiliary system that filters dust from the intake air to ensure stable engine operation; its reliability directly affects the performance and operational safety of the vehicle. Critically, the dust extraction impeller can exhibit severe erosion wear in extreme environments, but conventional degradation testing methods are costly and require considerable time to complete. Therefore, this study conducted accelerated degradation testing using the change in impeller blade thickness as the degradation indicator and the dust concentration and impeller rotational speed as dual elevated stress factors to obtain time-series degradation data from 48 blade samples. Linear, exponential, power-law, natural logarithmic, and Gompertz models were subsequently fit to the data for a single sample, and then the Technique for Order Preference by Similarity to Ideal Solution (TOPSIS) method was employed to select the optimal degradation trajectory model. The accuracy of the selected linear model was verified using the data from all samples, confirming that it can be applied to predict the degradation of the dust extraction impeller over time. The contribution of this study comprises the establishment of a degradation assessment framework combining accelerated degradation testing with TOPSIS-based model selection to provide a practical basis for the reliability design and maintenance planning of vehicle dust extraction impellers operating in extreme environments.

## 1. Introduction

During vehicle operation, the dust extraction impeller removes dust particles from the incoming air to prevent contaminants from entering the engine intake system. The impeller rotates at high speed in extremely harsh operating conditions, including the continuous impact of airborne sand and dust particles. This leads to severe erosion wear, reduced dust extraction efficiency, increased vibration and noise, and eventual blade fracture, all of which adversely affect normal vehicle operation [1,2]. Previous studies have shown that the particle trajectory, impact velocity, angle, and concentration are key factors influencing the severity of impeller erosion [3,4]. Recent studies have employed computational fluid dynamics and discrete element modeling to demonstrate that particle–flow interactions and particle collisions significantly affect the erosion distribution and wear evolution in turbomachinery components [5]. The severity of erosive wear is also influenced by environmental and material-related factors. Temperature and humidity can affect both the mechanical properties of blade materials and the interaction between airborne particles and blade surfaces, thereby influencing the wear rate. Furthermore, the chemical activity of airborne particles can accelerate material degradation through coupled erosion–corrosion effects. Thus, the erosion resistance of an impeller blade is heavily dependent on its material properties, such as hardness and toughness, and a protective surface coating can significantly improve its wear resistance and extend its service life.

Indeed, erosive wear is a complex degradation process governed by the combined effects of particle characteristics, operating conditions, environmental factors, and material properties. For example, the dust disturbed by the tires of vehicles operating in desert environments has been shown to significantly accelerate material loss from impeller surfaces [6], and analyses of compressor blade wear under transient vehicle-acceleration conditions have revealed the dynamic characteristics of particle erosion [7,8]. Recent studies quantifying the sand erosion of alloy materials have confirmed that dust concentration and impact velocity are the dominant environmental factors governing the wear rate and determined that their coupling effect exhibits nonlinear synergistic characteristics [9]. Although computational fluid dynamics simulations and experimental methods have been widely used to predict impeller blade erosion locations and wear patterns [10], the long-term service life evaluation of vehicle dust extraction impellers remains challenging owing to their complex degradation mechanisms and the limited data describing them.

Accelerated degradation testing (ADT) is an effective method for obtaining degradation data for highly reliable products and has been widely applied to evaluate mechanical components such as engine blades and rolling bearings [11,12,13]. This method reproduces degradation processes similar to those occurring under normal conditions within a shorter time by applying elevated stress factors, such as higher temperatures or dust concentrations [14,15,16]. In contrast to traditional accelerated life testing, ADT focuses on continuous degradation measurements (e.g., thickness loss, surface roughening, mass loss, or efficiency decline) rather than failure time. As a result, multi-stress stepwise or constant-stress ADT schemes, reliability assessment models that consider generalized coupling effects, and optimization design methods based on the Tweedie exponential dispersion process have been developed in recent years [17,18]. These methods provide a theoretical foundation for addressing issues associated with the degradation of vehicle dust extraction impellers, in which particle erosion is the dominant wear mechanism.

Despite extensive research on erosion mechanisms and ADT techniques, few studies have evaluated the design of ADT, selection of degradation indicators, or modeling of multiple stress factor coupling for vehicle dust extraction impellers specifically; previous studies have primarily focused on general mechanical components, such as blades or impellers [19]. Critically, the process by which vehicle dust extraction impellers degrade exhibits an obvious regional, progressive, and random nature that is difficult to describe accurately using traditional single stress-factor or deterministic models. Therefore, this study identified the dominant degradation trajectory and characterized the average degradation trend of dust extraction impellers subjected to different accelerated stress conditions. Deterministic degradation trajectory models were adopted to provide a first-step description of the degradation process because recent reviews have indicated that this approach is particularly practical when conducting reliability assessments and remaining useful life predictions using a limited number of degradation samples [20].

Although the degradation modeling and TOPSIS method employed in this study are established techniques, the novelty of this work lies in their application to vehicle dust extraction impellers, which have rarely been addressed in studies of degradation testing methodologies and degradation trajectory modeling. Specifically, this study (1) developed a dual-stress ADT framework based on dust concentration and rotational speed, (2) established a degradation dataset comprising 48 impeller blade samples subjected to controlled erosion conditions, and (3) proposed a systematic model selection strategy using TOPSIS to identify the most suitable degradation trajectory for characterizing impeller wear. Together, these contributions provide a practical framework for the degradation assessment and reliability-oriented maintenance of vehicle dust extraction impellers.

## 2. Methods

### 2.1. ADT Design for Dust Extraction Impellers

By applying stress levels above those present under normal operating conditions, ADT accelerates performance deterioration, enables the rapid acquisition of degradation data, and facilitates the inference of reliability characteristics under normal conditions. Therefore, the dust extraction impeller failure mechanism was analyzed in this study to identify the elevated stress factors to be applied, and the experimental platform and program were designed accordingly to obtain time-series degradation data for subsequent analysis.

#### 2.1.1. Failure Mechanism Analysis

A vehicle dust extraction impeller operates under the combined effects of centrifugal load, airflow impact, and particle erosion [21]. Its dominant failure mode is blade thinning caused by wear that exhibits regional concentration and gradual progression. This wear is primarily concentrated at the leading edge of the blade and the junction between the blade and rear plate, where the particles first impact at near-normal angles. The impeller failure mechanism involves both material damage and mechanical responses [22,23]. At the material level, particle impacts cause plastic deformation forming micropits and scratches that accumulate and develop into material spalling. At the mechanical level, stress concentrations occur in high-curvature regions during high-speed rotation, initiating fatigue cracks.

Therefore, the primary stress factors affecting the service life of a dust extraction impeller comprise the dust concentration and rotational speed. The dust concentration reflects the quantity of solid particles in the airflow impacting the blade surfaces at a given velocity, resulting in abrasive wear; the rotational speed affects both the centrifugal stress in and particle impact velocity on the blades. Notably, these two factors are coupled, and the combination of a high dust concentration and high rotational speed produces synergistic wear that accelerates impeller degradation.

#### 2.1.2. Experimental Platform

**Figure 1:**
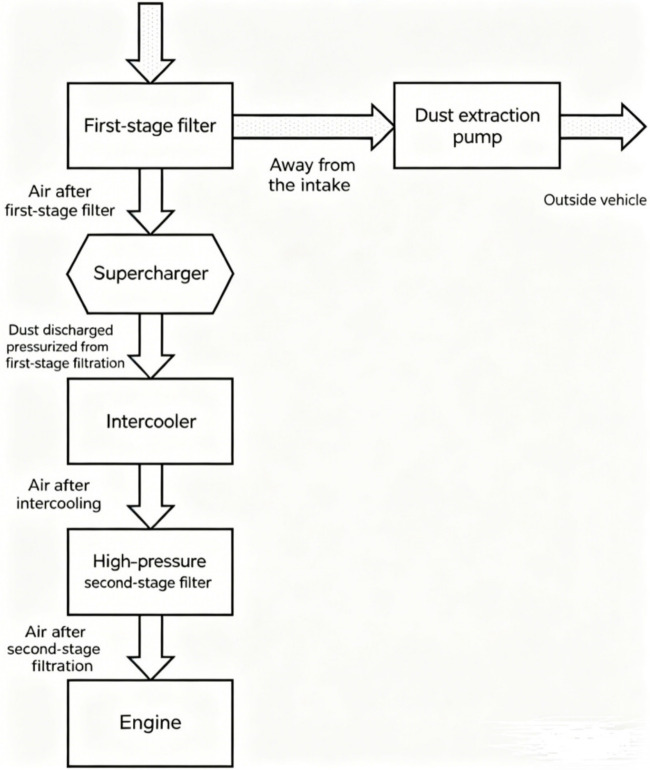
Schematic diagram showing the operation of the dust extraction pump in the engine air intake system.

**Figure 2:**
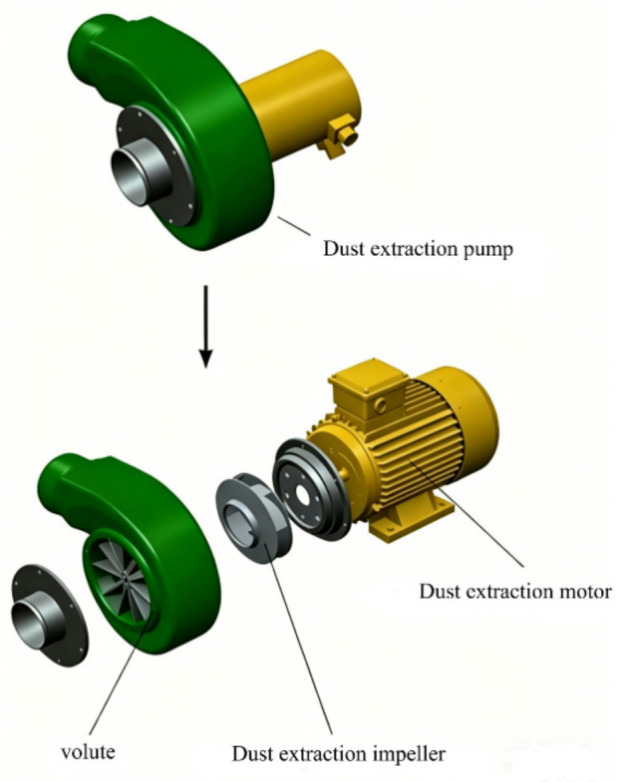
Exploded view showing the components of the dust extraction pump.

The vehicle dust extraction pump employs a rotary dust removal mechanism in which a motor rotates an impeller at high speed to pull the dust-laden airflow away from the first-stage filter and thereby complete the dust extraction process. The operation of the dust extraction pump within the engine air intake system is illustrated in Figure 1; the components of the dust extraction pump are shown in Figure 2.

**Figure 3:**
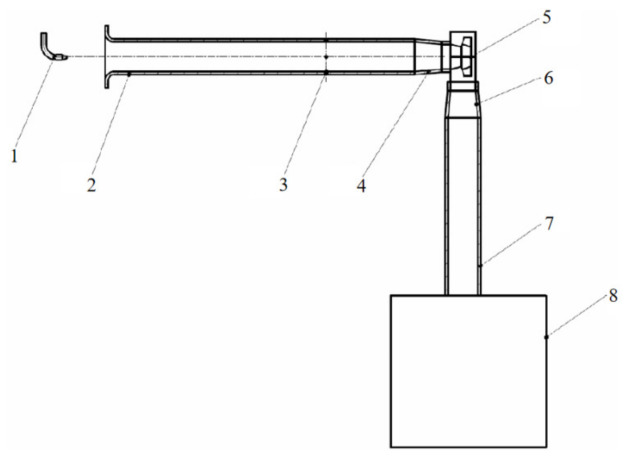
Schematic diagram of the dust extraction impeller blade wear test platform. 1—Dust injector. 2—Cylindrical air intake duct. 3—Static pressure measurement point for the extraction pump air intake. 4—Conical transition section between the air intake duct and extraction pump. 5—Dust extraction pump. 6—Conical transition section between the extraction pump and exhaust duct. 7—Cylindrical exhaust duct. 8—Dust collection device.

Figure 3 shows the ADT platform constructed in this study based on the operation of the dust extraction pump within the engine air intake system. The impeller was installed on a motor assembly to create the dust extraction pump. The dust injector continuously delivered ultra-fine quartz sand (particle size: 270 mesh) at the desired concentration to the extraction pump through the cylindrical air intake duct, where the static pressure at the pump inlet was monitored at point 3. The dust-laden flow subsequently passed through the conical transition section into the pump, and then exited through the conical transition section and cylindrical exhaust duct. The dust collection device captured the dust expelled by the extraction pump through the exhaust duct. The blade thickness loss, defined as the difference between the initial and current impeller blade thicknesses, was used as the performance indicator because it directly affects the dust extraction efficiency and engine air intake quality.

#### 2.1.3. Experimental Program

The typical operating conditions of the considered vehicle dust extraction impeller are listed in Table 1. Based on the analysis presented in Section 2.1.2, the blade thickness degradation trends were obtained under the different elevated dust concentrations and rotational speeds listed in Table 2. Therefore, the ADT process evaluated 12 dust extraction impellers under different accelerated stress conditions. Four blades from each impeller were selected for thickness measurements throughout the test, resulting in a total of 48 degradation samples for subsequent modeling and validation.

**Figure 4:**
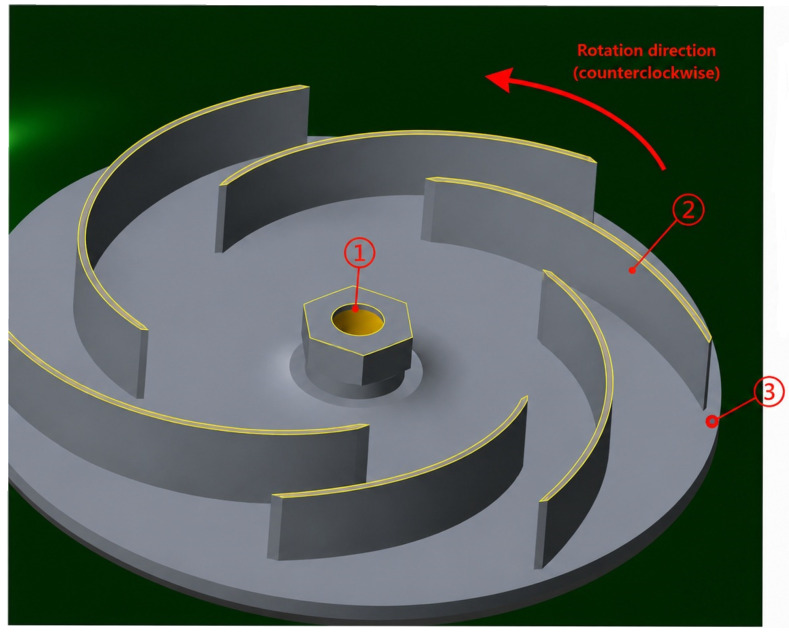
Diagram showing the blade thickness measurement point on the dust extraction impeller. 1—Hub (shaft connection). 2—Impeller blade. 3—Measurement point (blade tip).

Because the impeller rotates in the counterclockwise direction as the dust-laden airflow enters it axially through the inlet, and then discharges radially toward the outlet under the action of centrifugal force, erosion wear during degradation will be most pronounced at the blade tip. Therefore, the outermost position at the junction between the blade and weld to the rear circular plate was chosen as the blade thickness measurement point on each impeller, as shown in Figure 4.

Based on the industry standard for dust extraction pump testing, the ADT process was terminated when the thickness of any impeller blade decreased below 0.8 mm. This threshold was adopted as the practical failure criterion and provides an engineering basis for evaluating blade wear progression.

The blade thickness degradation (wear loss) over time was determined from the blade thickness measurements as follows:

(1) $$\Delta d=d_{0}-d_{1}$$

where $d_{0}$ is the initial blade thickness and $d_{1}$ is the blade thickness at the time of measurement.

**Figure 5:**
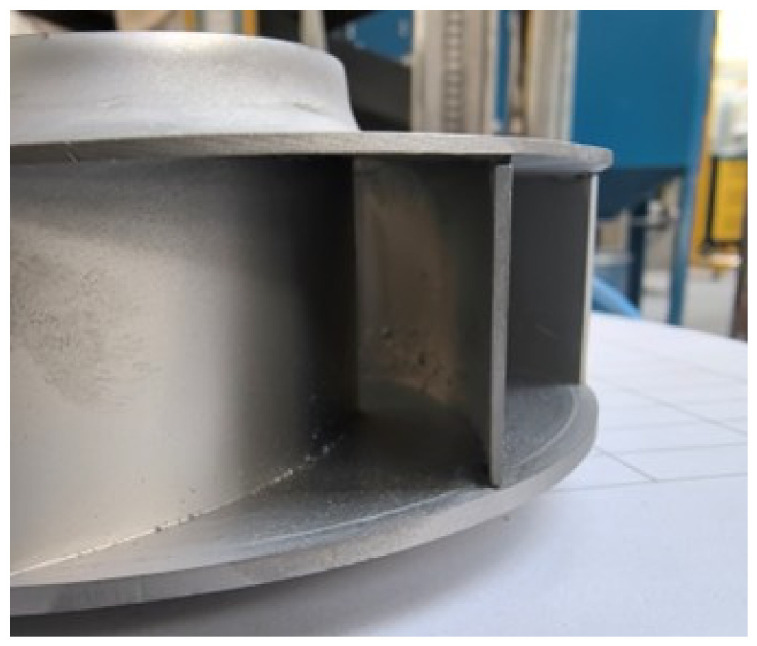
Wear condition of a representative impeller blade after ADT.

Figure 5 shows the wear condition of a representative impeller blade after ADT. Visible wear marks and local material loss can be observed on the blade surface that was exposed to continuous particle impact.

### 2.2. Degradation Trajectory Modeling

#### 2.2.1. Evaluated Degradation Models

**Figure 6:**
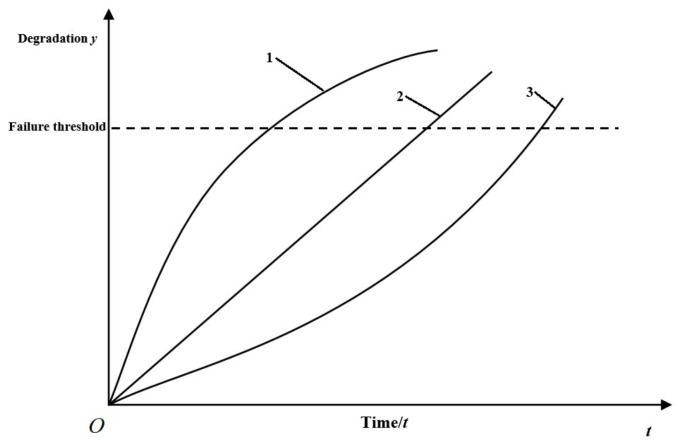
Types of degradation trajectories. 1—Convex degradation trajectory. 2—Liner degradation trajectory. 3—Concave degradation trajectory.

A degradation model is a mathematical framework used to quantify the pattern of decline in the performance of a system, device, or material over time under specific usage conditions. It captures the stochastic or deterministic characteristics of the degradation process by establishing a functional relationship between performance parameters and degradation factors. As shown in Figure 6, degradation trajectories can be classified as linear, concave, or convex depending on the rate of change in the extent of degradation over time [24].

In most cases, when permissible errors are ignored, a degradation trajectory can be fitted to degradation data using the linear, exponential, power-law, natural logarithmic, or Gompertz models defined in Table 3. Parametric degradation trajectory models are generally preferred when fitting the data obtained by ADT of mechanical components owing to their clear physical meaning, low sample requirement, and stable extrapolation performance [25]. These models describe the relationship between the product performance degradation parameter $y_{i}$ (i.e., the wear loss of the impeller blade in this study) and time. The resulting fitted trajectory is regarded as the actual degradation trajectory for that sample. The unknown parameters $\alpha_{i}$ and $\beta_{i}$ in each model were fitted to the experimental data in this study using a regression analysis.

#### 2.2.2. TOPSIS-Based Model Selection

**Figure 7:**
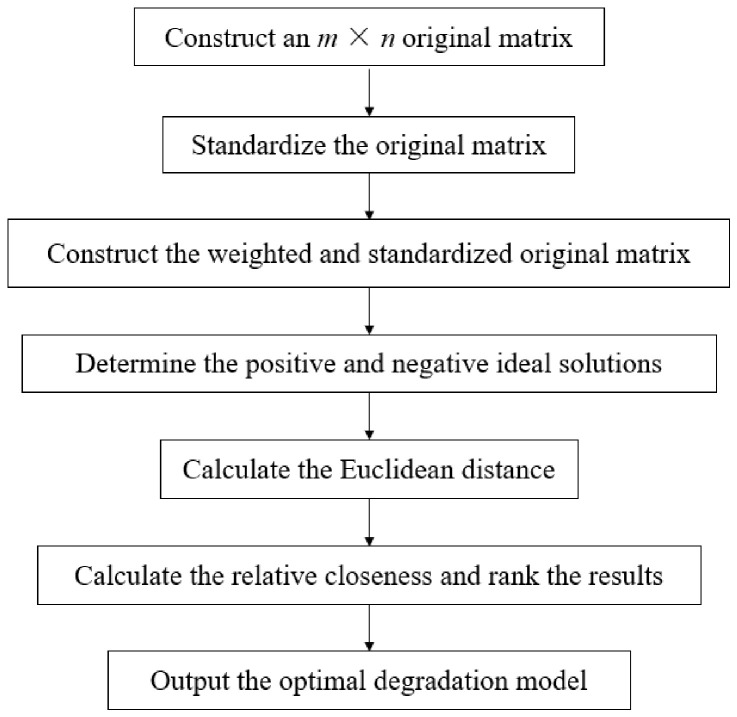
Flowchart of the decision-making process employed in the TOPSIS method.

This study employed the TOPSIS method to select the optimal impeller blade degradation model. Widely used as a multicriteria decision-making method, TOPSIS ranks alternatives by calculating their distances to ideal positive and negative solutions, and then identifies the optimal option according to its proximity to the positive solution, which reflects its quality [26]. A flowchart of the decision-making process employed in the TOPSIS method is shown in Figure 7, in which *m* denotes the number of candidate degradation models and *n* denotes the number of evaluation criteria. In this study, the candidate degradation models comprised the linear, exponential, power-law, natural logarithmic, and Gompertz models, and the evaluation criteria comprised the sums of the squared fitting errors ($S_{SE}$ values) obtained under different dust concentrations.

The process applied in this study to select the optimal degradation trajectory model using the TOPSIS method comprised six steps.

Step 1: Assemble the attribute data for the considered models, defined in this study as the $S_{SE}$ between each model and the experimental data, to construct the original matrix (***X***) given by

(2) $$X=\left[\begin{array}{cccc} x_{11} & x_{12} & \ldots & x_{1n}\\ x_{21} & x_{22} & \ldots & x_{2n}\\ \vdots & \vdots & \ddots & \vdots \\ x_{m1} & x_{m2} & \ldots & x_{mn} \end{array}\right]$$

Step 2: Eliminate the effects of different units across attributes by standardizing the original matrix using the range normalization method as follows:

(3) $$Z={\left(z_{ij}\right)}_{m\times n}$$

where $z_{ij}$ represents the normalized attribute value satisfying 0 ≤ $z_{ij}$ ≤ 1.

Step 3: Calculate the weighted normalized matrix by combining *Z* with the attribute weights $w_{j}$ to obtain the weighted normalized matrix $V={\left(v_{ij}\right)}_{m\times n}$, in which:

(4) $$v_{ij}=z_{ij}\times w_{j}$$

where $v_{ij}$ is the weighted normalized attribute value and $w_{j}=\frac{1}{n}$ in this study.

Step 4: Determine the ideal positive and negative solutions. The ideal positive solution is a hypothetical alternative composed of the best value for each attribute and is expressed as

(5) $$V^{+}=\left\{v_{1}^{+},v_{2}^{+},\ldots ,v_{n}^{+}\right\}$$

where $v_{j}^{+}=\max_{i=1,\ldots ,m}v_{ij}$.

The ideal negative solution is a hypothetical alternative composed of the worst value for each attribute and is expressed as

(6) $$V^{-}=\left\{v_{1}^{-},v_{2}^{-},\ldots ,v_{n}^{-}\right\}$$

where $v_{j}^{-}=\min_{i=1,\ldots ,m}v_{ij}$.

Step 5: Calculate the Euclidean distances from each alternative to the ideal solutions. The distance from alternative $A_{i}$ to the ideal positive solution is given by:

(7) $$D_{i}^{+}=\sqrt{\sum_{j=1}^{n}{\left(v_{ij}-v_{j}^{+}\right)}^{2}}$$

and the distance from alternative $A_{i}$ to the ideal negative solution is given by:

(8) $$D_{i}^{-}=\sqrt{\sum_{j=1}^{n}{\left(v_{ij}-v_{j}^{-}\right)}^{2}}$$

Step 6: Calculate the relative closeness $C_{i}$ for each alternative Ai and rank the alternatives accordingly. The value of $C_{i}$ represents the degree to which alternative Ai approaches the ideal positive solution and is calculated by

(9) $$C_{i}=\frac{D_{i}^{+}}{D_{i}^{+}+D_{i}^{-}}$$

Equation (9) indicates that when $C_{i}=1$, alternative Ai corresponds to the ideal positive solution (i.e., the optimal model), whereas when $C_{i}=0$, alternative Ai corresponds to the ideal negative solution (i.e., the least favorable model). Thus, the relative quality of each model can be determined by ranking the alternatives in order of descending $C_{i}$.

## 3. Results

### 3.1. Experimental Data

**Figure 8:**
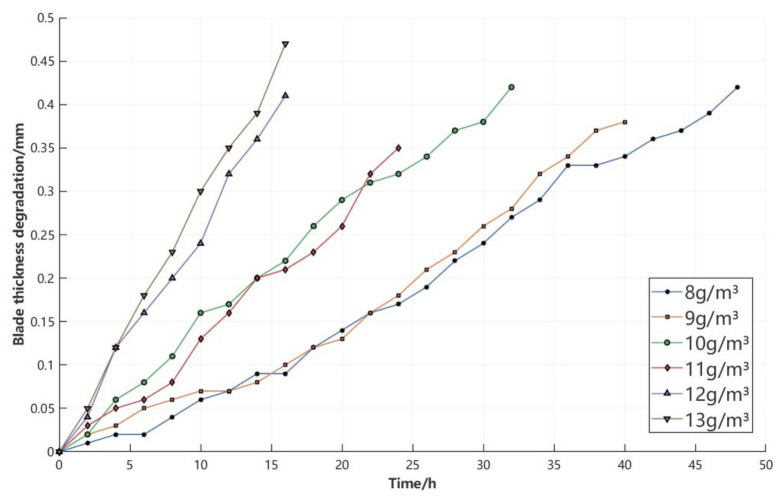
Example blade thickness degradation curves for different dust concentrations at 16,000 rpm.

**Figure 9:**
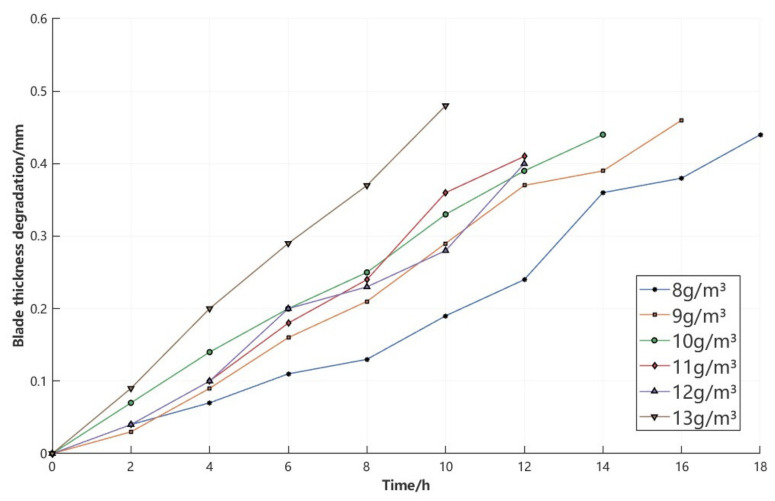
Example blade thickness degradation curves for different dust concentrations at 18,000 rpm.

Four blade samples were measured during ADT under each stress condition. The measured blade thickness degradation (wear loss) values over time are plotted for one representative blade sample under each evaluated dust concentration at the two considered rotational speeds in Figure 8 and Figure 9. The degradation data for all 48 blade samples were used in the subsequent model fitting and validation analyses.

As shown in Figure 8 and Figure 9, blade thickness degradation became more severe as the dust concentration increased at both rotational speeds. In addition, a comparison of the degradation curves obtained at 16,000 and 18,000 rpm indicated that the higher rotational speed generally accelerated the wear process. These observations confirm that both stress factors exerted significant influence on the degradation behavior observed during ADT.

### 3.2. Model Fitting

The thickness degradation data comprising the four thickness measurements for blade 1 under different dust concentrations at an impeller rotational speed of 16,000 rpm were used to determine the unknown fitting parameters for the five degradation trajectory models; the results are shown in Table 4. Note that the primary objective of this study was to identify the most suitable degradation trajectory model for vehicle dust extraction impellers under accelerated erosion conditions, not to establish a unified stress-coupled degradation equation. Therefore, the effects of dust concentration and rotational speed were considered solely through the stress levels applied in the ADT.

The fitted parameters listed in Table 4 varied systematically with the applied stress levels. For example, the slope parameter, $\alpha_{i}$, of the linear model generally increased with the dust concentration, indicating a higher degradation rate under more severe erosion conditions. Thus, although dust concentration and rotational speed were not explicitly included as variables in a single predictive equation, their effects are quantitatively reflected in the degradation data and corresponding fitted model parameters obtained under different accelerated stress conditions.

**Figure 10:**
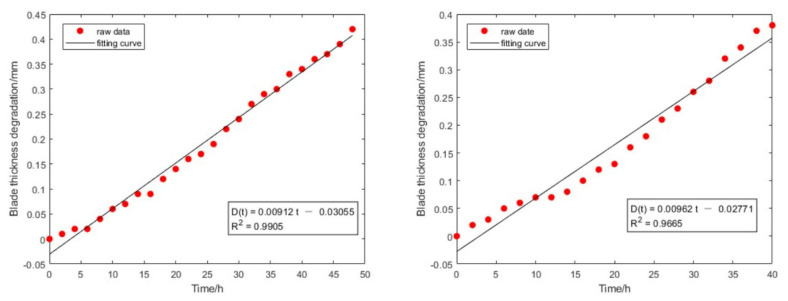
Linear model curves of blade 1 thickness degradation over time for dust concentrations of 8 g/m^3^ (**left**) and 9 g/m^3^ (**right**) at 16,000 rpm.

**Figure 11:**
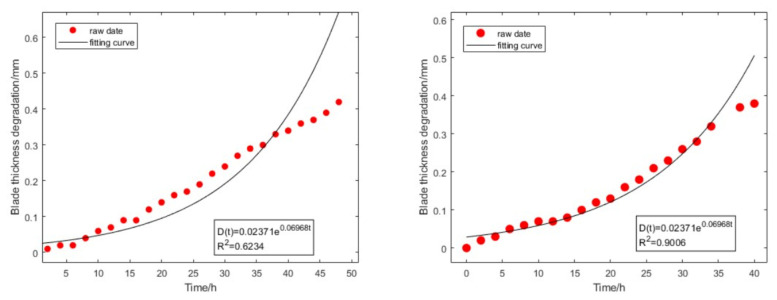
Exponential model curves of blade 1 thickness degradation over time for dust concentrations of 8 g/m^3^ (**left**) and 9 g/m^3^ (**right**) at 16,000 rpm.

**Figure 12:**
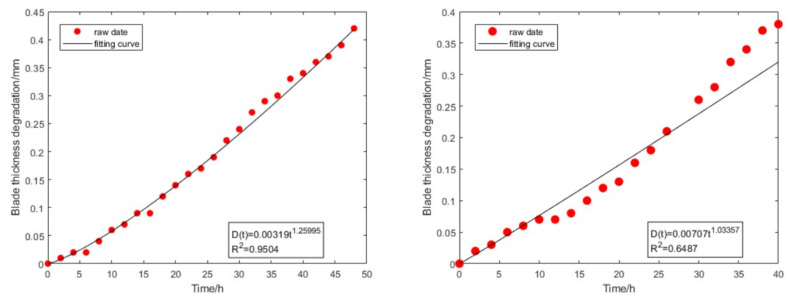
Power-law model curves of blade 1 thickness degradation over time for dust concentrations of 8 g/m^3^ (**left**) and 9 g/m^3^ (**right**) at 16,000 rpm.

**Figure 13:**
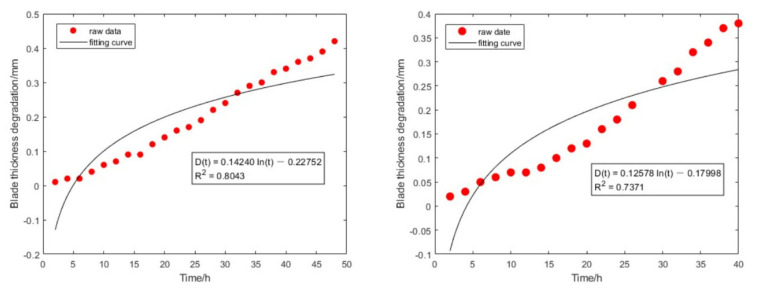
Logarithmic model curves of blade 1 thickness degradation over time for dust concentrations of 8 g/m^3^ (**left**) and 9 g/m^3^ (**right**) at 16,000 rpm.

**Figure 14:**
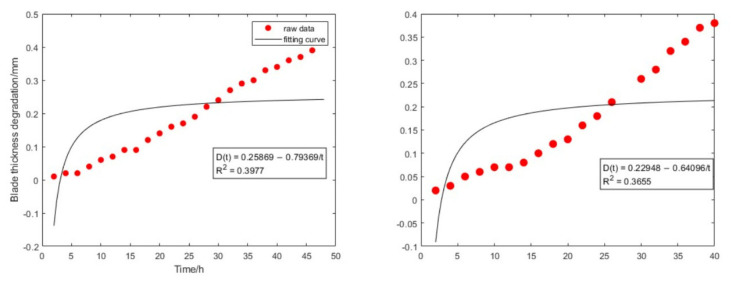
Gompertz model curves of blade 1 thickness degradation over time for dust concentrations of 8 g/m^3^ (**left**) and 9 g/m^3^ (**right**) at 16,000 rpm.

The blade degradation trajectory curves fitted to the data using the five models for blade 1 at 16,000 rpm are shown in Figure 10, Figure 11, Figure 12, Figure 13 and Figure 14 (only the curves for dust concentrations of 8 and 9 g/m^3^ are presented for simplicity). These curves show that the blade thickness wear loss progressively increased throughout the duration of the test, and the degradation rate accelerated as the dust concentration increased.

### 3.3. Optimal Model Selection

The $S_{SE}$ results for the five thickness degradation models for blade 1 at 16,000 rpm are presented in Table 5.

The original matrix for blade 1 was constructed based on Equation (2) using the $S_{SE}$ values in Table 5 as follows:

(10) $$X=S_{SE}=\left[\begin{array}{ccccc} 0.004137 & 0.150621 & 0.001863 & 0.078279 & 0.240896\\ 0.009887 & 0.023589 & 0.017731 & 0.069200 & 0.167005\\ 0.002294 & 0.072359 & 0.004890 & 0.019330 & 0.090697\\ 0.002927 & 0.021367 & 0.003691 & 0.028869 & 0.081682\\ 0.000916 & 0.018054 & 0.001341 & 0.008707 & 0.030519\\ 0.000516 & 0.022754 & 0.000883 & 0.009931 & 0.038437 \end{array}\right]$$

Next, the TOPSIS method described in Section 2.2.2 was applied to calculate the relative closenesses $C_{i}$ of the five degradation models as follows:

(11) $$C_{i}=\left[\begin{array}{ccccc} 0.957053 & 0.197846 & 0.908235 & 0.308904 & 0.053274 \end{array}\right]$$

**Figure 15:**
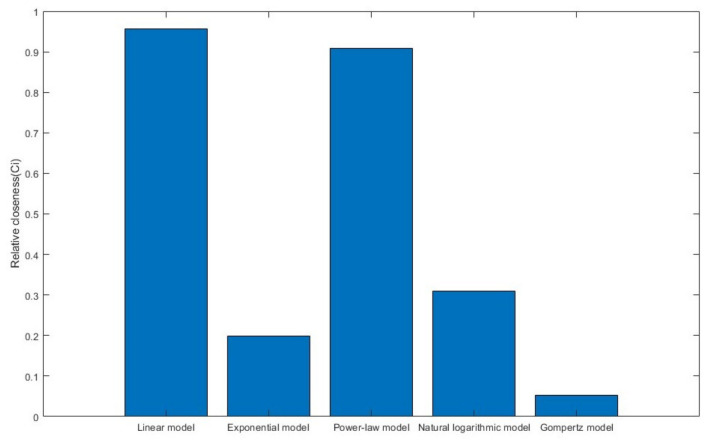
Relative closenesses of the five models for blade 1.

The $C_{i}$ values for the five models are compared in Figure 15; the closer the value of $C_{i}$ is to 1, the closer the corresponding model is to the optimal solution.

Figure 15 shows that $C_{1}$ was closest to 1, indicating that the linear degradation model provided the best fit to the blade 1 data. Therefore, the linear model was selected as the optimal approach for modeling the degradation of dust extraction impeller blades.

Note that blade 1 was selected as a representative example in this analysis to illustrate the degradation model fitting and TOPSIS-based model selection procedure. The selected blade sample was drawn from a dataset containing blade thickness measurements for 48 samples from 12 dust extraction impellers subjected to different accelerated stress conditions. The purpose of this analysis was to demonstrate the model selection methodology rather than to establish conclusions based solely on the analyzed blade data. The generality of the selected degradation model is evaluated using all 48 blade samples in Section 3.4.

### 3.4. Evaluation of the Selected Model

Finally, the robustness and general applicability of the selected degradation model was validated using blade thickness measurements for all 48 samples from the 12 tested dust extraction impellers. These samples reflect the effects of different dust concentrations, rotational speeds, and blade-to-blade variations, thereby providing a broad basis for evaluating the applicability of the selected degradation trajectory model. The linear model equation presented in Table 3 was used and a regression analysis was performed on the degradation data from all 48 samples obtained under different elevated stress factors to determine the fitting parameters $\alpha_{i}$ and $\beta_{i}$ for each sample. The correlation coefficient *R*^2^ was subsequently calculated to evaluate the goodness of fit of each resulting model. The fitting parameter values and corresponding *R*^2^ coefficients for the linear degradation trajectory models are listed in Table 6.

The fitting results indicate that the *R*^2^ values obtained for all 48 linear degradation models exceeded 0.94 despite variations in dust concentration, rotational speed, and individual blade characteristics. This confirms that the linear model effectively described the blade thickness degradation of all samples over time and indicates its suitability for accurately modeling the degradation trajectories of dust extraction impellers. Furthermore, the robustness of the model selected using the TOPSIS procedure against variations in wear conditions supports the rationality of the TOPSIS-based model selection process. Indeed, although variations among the fitting parameters for the 48 blade samples reflect sample-to-sample variability, the overall degradation behavior was effectively described by the selected linear trajectory model. Therefore, this degradation model provides an excellent foundation for predicting the lifespan and assessing the reliability of vehicle dust extraction impellers.

## 4. Conclusions

This study addressed the difficulty, long duration, and high costs of assessing the lifespans of vehicle dust extraction impellers in extreme environments by systematically investigating impeller degradation using ADT, modeling the obtained degradation data using different equations, and selecting an optimal degradation trajectory model using TOPSIS. The primary conclusions and contributions of this research are summarized as follows:

(1) A dual-stress-factor ADT approach was designed to obtain reliable impeller degradation data. Considering the actual operating conditions and failure mechanisms of dust extraction impellers, the dust concentration and impeller rotational speed were identified as the key stress factors for evaluation, and the blade thickness loss was used as the performance degradation indicator. An ADT scheme applying two rotational speeds and six dust concentrations was subsequently implemented to obtain time-series blade thickness degradation data from 48 blade samples for subsequent modeling.
(2) The TOPSIS multi-criteria decision-making method was used to select the optimal degradation trajectory model from among linear, exponential, power-law, logarithmic, and Gompertz models fitted to the experimental data. The linear model was determined to represent the optimal degradation trajectory model and was applied to determine the blade thickness degradation for each sample. The results indicated suitable agreement between the results obtained by the experiments and linear model, confirming its ability to predict dust extraction impeller degradation. The results also demonstrated that both the dust concentration and rotational speed significantly influenced the impeller blade degradation rate. This effect is supported by the degradation trends observed under different stress conditions as well as the systematic variation in the fitted model parameters. Therefore, the accelerated stress factors were successfully incorporated into the degradation assessment framework through the experimental design and parameter identification process.

The results of this study provide data support and a theoretical framework for the reliability design, predictive maintenance strategy development, and service life assurance of dust extraction impellers operating in complex and extreme environments, such as those encountered by specialized vehicles. Future research should incorporate stochastic Wiener processes or deep learning models (e.g., the long short-term memory model) to account for individual differences among samples and enable online remaining useful life prediction. These models could also be validated against actual vehicle operating profiles to enhance their prediction accuracy and practical engineering applicability.

Note that the contribution of this study is not the development of a new degradation model, but the establishment of a practical degradation assessment framework for vehicle dust extraction impellers. Although deterministic degradation models were considered in this study, future work will investigate stochastic degradation approaches to further characterize uncertainty and sample-to-sample variability in impeller degradation. In addition, independent validation datasets or cross-validation procedures were not considered in the present study and will be investigated in future work.

The degradation trajectory model identified in this study provides a basis for the reliability-oriented evaluation of dust extraction impellers. The degradation state of an impeller can be monitored and extrapolated to estimate its remaining useful life by combining the fitted degradation model with the failure threshold defined in this study. Furthermore, the degradation information obtained from ADT can support the assessment of reliability under different operating conditions and assist maintenance personnel in determining appropriate inspection and replacement intervals. Therefore, the proposed degradation modeling framework shows promise for application in the condition-based and predictive maintenance of vehicle dust extraction systems.

## Figures and Tables

**Table 1:** Typical operating conditions of the dust extraction impeller.

Dust Concentration	Rotational Speed	Temperature	Humidity
3–4 g/m^3^	12,000 rpm	−50–80 °C	40–60%

**Table 2:** Primary experimental conditions evaluated in this study.

Experimental Condition	Applied Values
Elevated stress factor 1 (dust concentration)	Ranging from 8–13 g/m^3^ in increments of 1 g/m^3^
Elevated stress factor 2 (impeller rotational speed)	16,000 or 18,000 rpm
Blade thickness measurement rate and test time	Every 2 h

**Table 3:** Common degradation models considered in this study.

Model	Equation
Linear [24]	$y_{i}=\alpha_{i}t+\beta_{i}$
Exponential [24]	$y_{i}=\alpha_{i}e^{\beta_{i}t}$
Power-law [24]	$y_{i}=\alpha_{i}t^{\beta_{i}}$
Natural logarithmic [24]	$y_{i}=\alpha_{i}\ln t+\beta_{i}$
Gompertz [26]	$y_{i}=\alpha_{i}-\frac{\beta_{i}}{t}$

**Table 4:** Estimated fitting parameters for the five degradation trajectory models for blade 1 at 16,000 rpm.

Dust Concentration (g/m^3^)	Fitting Parameter	Linear Model	Exponential Model	Power-Law Model	Natural Logarithmic Model	Gompertz Model
8	$\alpha_{i}$	0.0091	0.023714	0.003186	0.1424	0.2587
	$\beta_{i}$	−0.0306	0.069679	1.259954	−0.2275	0.7937
9	$\alpha_{i}$	0.0096	0.0289	0.0071	0.1258	0.2295
	$\beta_{i}$	−0.0277	0.0716	1.0336	−0.1800	0.6410
10	$\alpha_{i}$	0.0130	0.0493	0.0124	0.1497	0.3160
	$\beta_{i}$	0.0098	0.0778	1.0367	−0.1588	0.7964
11	$\alpha_{i}$	0.0148	0.0360	0.0116	0.1408	0.2708
	$\beta_{i}$	−0.0157	0.1010	1.0494	−0.1518	0.6606
12	$\alpha_{i}$	0.0257	0.0526	0.0223	0.1723	0.3556
	$\beta_{i}$	0.0002	0.1431	1.0636	−0.1165	0.7322
13	$\alpha_{i}$	0.0290	0.0603	0.0261	0.1950	0.4017
	$\beta_{i}$	0.0002	0.1419	1.0474	−0.1325	0.8269

**Table 5:** *S*_SE_ values for the five degradation models for blade 1 at 16,000 rpm.

Dust Concentration (g/m^3^)	Linear Model	Exponential Model	Power-Law Model	Natural Logarithmic Model	Gompertz Model
8	0.004137	0.150621	0.001863	0.078279	0.240896
9	0.009887	0.023589	0.017731	0.069200	0.167005
10	0.002294	0.072359	0.004890	0.019330	0.090697
11	0.002927	0.021367	0.003691	0.028869	0.081682
12	0.000916	0.018054	0.001341	0.008707	0.030519
13	0.000516	0.022754	0.000883	0.009931	0.038437

**Table 6:** Fitting parameters and correlation coefficients (*R*^2^) for the linear degradation trajectory models.

Sample	$\alpha_{i}$	$\beta_{i}$	*R* ^2^	Sample	$\alpha_{i}$	$\beta_{i}$	*R* ^2^
1	0.009123	−0.03055	0.9905	25	0.02497	−0.02873	0.968
2	0.008131	−0.01314	0.9884	26	0.02203	−0.02327	0.9804
3	0.008262	−0.03028	0.9881	27	0.02482	−0.05036	0.9465
4	0.009242	−0.02022	0.9947	28	0.02555	−0.04291	0.9751
5	0.009623	−0.02771	0.9665	29	0.03008	−0.01844	0.9918
6	0.01152	−0.0461	0.9775	30	0.031	−0.03356	0.9813
7	0.008961	−0.009221	0.9925	31	0.0295	−0.03378	0.9771
8	0.009526	−0.02528	0.9872	32	0.02492	−0.02822	0.9848
9	0.01303	0.009804	0.9918	33	0.03155	0.006667	0.9982
10	0.0127	−0.01137	0.994	34	0.0294	−0.0008333	0.9993
11	0.01152	−0.00549	0.9945	35	0.03202	0.0008333	0.9914
12	0.01397	−0.01176	0.9946	36	0.0297	0.01333	0.99
13	0.0149	−0.01657	0.9843	37	0.03589	−0.02536	0.9843
14	0.01755	−0.03171	0.9857	38	0.03518	−0.0025	0.9837
15	0.0152	−0.02114	0.9825	39	0.03554	0.001071	0.9972
16	0.01575	0.01029	0.9899	40	0.03625	−0.02893	0.982
17	0.02567	0.0002222	0.9942	41	0.03232	−0.01536	0.9774
18	0.02492	−0.001556	0.9844	42	0.04036	−0.04357	0.977
19	0.02733	−0.003111	0.9971	43	0.0375	0.002143	0.9912
20	0.02783	−0.02822	0.9774	44	0.03929	−0.01571	0.979
21	0.029	0.0002222	0.9975	45	0.04757	0.0004762	0.9983
22	0.02917	−0.002222	0.9926	46	0.04557	0.0004762	0.9965
23	0.02842	−0.01622	0.9904	47	0.04614	0.007619	0.9957
24	0.03142	−0.03244	0.9793	48	0.049	−0.01333	0.9964

## Data Availability

The original contributions presented in this study are included in the article. Further inquiries can be directed to the corresponding author.
